# Flavonoids from *Tetracera indica* Merr. induce adipogenesis and exert glucose uptake activities in 3T3-L1 adipocyte cells

**DOI:** 10.1186/s12906-017-1929-3

**Published:** 2017-08-30

**Authors:** Md. Mahmudul Hasan, Qamar Uddin Ahmed, Siti Zaiton Mat Soad, Jalifah Latip, Muhammad Taher, Tengku Muhamad Faris Syafiq, Murni Nazira Sarian, Alhassan Muhammad Alhassan, Zainul Amiruddin Zakaria

**Affiliations:** 1 Department of Pharmaceutical Chemistry, Kulliyyah of Pharmacy, International Islamic University Malaysia, Pahang DM, 25200 Kuantan, Malaysia; 2 Department of Pharmaceutical Technology, Kulliyyah of Pharmacy, International Islamic University Malaysia, Pahang DM, 25200 Kuantan, Malaysia; 3 School of Chemical Sciences and Food Technology, Faculty of Science and Technology, Universiti Kebangsaan Malaysia, 43600 Bandar Baru Bangi, Selangor Malaysia; 4 Halal Institute Research Institute, Universiti Putra Malaysia, 43400 Serdang, Selangor Malaysia

**Keywords:** *Tetracera indica* Merr., Flavonoids, 3T3-L1 preadipocyte cells, Adipogenesis, 2-NBDG glucose uptake activity, Insulin like activity, Insulin sensitizing activity

## Abstract

**Background:**

*Tetracera indica* Merr. (Family: Dilleniaceae), known to the Malay as ‘*Mempelas paya*’, is one of the medicinal plants used in the treatment of diabetes in Malaysia. However, no proper scientific study has been carried out to verify the traditional claim of *T. indica* as an antidiabetic agent. Hence, the aims of the present study were to determine the in vitro antidiabetic potential of the *T. indica* stems ethanol extract, subfractions and isolated compounds.

**Methods:**

The ethanol extract and its subfractions, and isolated compounds from *T. indica* stems were subjected to cytotoxicity test using MTT viability assay on 3T3-L1 pre-adipocytes. Then, the test groups were subjected to the in vitro antidiabetic investigation using 3T3-L1 pre-adipocytes and differentiated adipocytes to determine the insulin-like and insulin sensitizing activities. Rosiglitazone was used as a standard antidiabetic agent. All compounds were also subjected to fluorescence glucose (2-NBDG) uptake test on differentiated adipocytes. Test solutions were introduced to the cells in different safe concentrations as well as in different adipogenic cocktails, which were modified by the addition of compounds to be investigated and in the presence or absence of insulin. Isolation of bioactive compounds from the most effective subfraction (ethyl acetate) was performed through repeated silica gel and sephadex LH-20 column chromatographies and their structures were elucidated through ^1^H–and ^13^C–NMR spectroscopy.

**Results:**

Four monoflavonoids, namely, wogonin, norwogonin, quercetin and techtochrysin were isolated from the *T. indica* stems ethanol extract. Wogonin, norwogonin and techtochrysin induced significant (*P* < 0.05) adipogenesis like insulin and enhanced adipogenesis like rosiglitazone. Wogonin and norwogonin also exhibited significant (*P* < 0.05) glucose uptake activity.

**Conclusion:**

The present study demonstrated that the flavonoids isolated from the *T. indica* stems possess antidiabetic potential revealing insulin-like and insulin-sensitizing effects which were significant among the compounds. This also rationalizes the traditional use of *T. indica* in the management of diabetes in Malaysia.

## Background

Diabetes mellitus (DM) is a very old disease which was reported by the ancient Egyptians more than 300 years ago. Diabetes is a metabolic disorder which is caused by partial or complete absence of insulin or insulin resistance. At present time, occurrence of diabetes has become a major global health issue because of its alarming prevalence all over the world. It is projected that about 20% of the world’s population is liable to become diabetic over the next 10 years. According to the Center for Disease Control and Prevention (CDC, USA), 29.1 million people in the United States of America have diabetes, which is 9.3% of their total population. World’s major pharmaceutical industries are in a race to fight against the chronic complications of diabetes and are coming up with different forms of insulin and insulin mimetics [1].

For type-2 diabetes mellitus (T2DM), several types of therapeutic approaches are taken into account, viz., improving insulin secretion by the pancreas, ameliorating the sensitivity of insulin to target receptor or increasing glucose uptake in adipocyte cells. In recent times, research works on adipocytes have been growing immensely. In vitro research studies for insulin and insulin mimetics exploit the concept to use glucose uptake method using adipocytes for these bioassays. Consequently, adipocytes have been emerging as an important drug target for diabetes and obesity-mediated metabolic syndrome. Adipose tissue, or fat, is an anatomical term for loose connective tissue composed of adipocytes, is not only known for its capability to store the excess of dietary energy in the form of triglycerides, but has also been regarded to play a key role in the regulation of energy metabolism. Adipogenesis is a complex process whereby pre-adipocytes become mature adipocytes with hundreds of genes alterations in the presence of insulin. A number of transcriptional factors have been widely recognised as being involved in the manifestation of adipogenesis, glucose uptake, and glycolysis pathway. These transcription factors mainly include adipokines for example leptin, glucose transporter-4 (GLUT4) and peroxisome proliferator-activated receptor-γ (PPARγ). A morphological alteration from fibroblastic to spherical occurs in the very first step of the adipogenesis when the alteration of the cell shape is accompanied by changes in the level of cytoskeletal and extra cellular matrix (ECM) components [2]. Morphological change of cells, gene expression and lipid accumulation occur gradually by the proteolytic reaction of the stromal ECM [3]. Finally, an elevated enzymatic activity in protein and mRNA level take place which includes the increased insulin sensitivity as well as the glucose transporters [4]. Hence, these transcription factors serve as model systems for evaluating the differentiating program in normal conditions or in conditions of diabetes, insulin resistance and obesity [5].

The search for a new class of safe antidiabetic agents is regarded as an important scientific endeavour to overcome chronic diabetes and its related infirmities. Therefore, there have always been continuous searches for alternative drugs. Ethnomedicinal plants have been recognised as the best source to obtain a variety of drugs according to the World Health Organization [6]. With respect to discard the hypoglycaemic side effect along with other deleterious effects of synthetic drugs, multitudinous ethnomedicinal plants are considered as the point of interest to ethno-botanical community as they have been proved to exhibit important medicinal properties including hypoglycaemic and anti-hyperglycaemic effects [7]. In this regard, many ethnomedicinal plants have been subjected to numerous scientific investigations and have proved extremely beneficial in providing medicinally active agents with desired pharmacological properties to treat the ailments such as type-1 DM and type-2 DM [8].

Presently, scientists have become more fascinated towards ethnomedicinal plants around the world and a numerous biological studies are currently undergoing in regard to discover safe and effective antidiabetic agents from natural sources. In this regard, *Tetracera indica* (Houtt. Ex Christm. & Panz.) Merr. (Dilleniaceae) is one of the Malaysian medicinal plants to address this issue effectively. It is a large, woody, rain forest climber of Malaysia which is commonly known as Mempelas paya or sand paper plant. It has white coloured flowers and leaves are simple and medium shaped. It has berry-like fruits which are sour in taste [9]. Different parts of the *T. indica* Merr. have been used for healing fever, flu, sinus symptoms, skin rashes, itching, piles, mouth ulcer, diarrhoea, insects bites and diabetes. *T. indica* is also used as one of the active ingredients in a local herbal drug viz., Plantisol®, which is widely prescribed and recommended to effectively manage diabetes in Malaysia by the local herbalist practitioners. *Barringtonia racemosa, Pithecellobium jiringa, Tinospora crispa* and *Andrographis paniculata* are the other active ingredients of Plantisol® [10]. The in vitro antidiabetic investigation of the aqueous extract of *T. indica* leaves has already been reported to decrease triglycerides accumulation on 3T3-L1 cells in a dose-dependent manner whereas cells treated with methanol extract significantly induced lipid accumulation. Moreover, both the polar extracts have been further reported to exhibit significant bioactivity in the in vitro 2-deoxy-D-[3H] glucose uptake test which proves its potential of being an antidiabetic agent [11]. However, active principles responsible for its antidiabetic effect are yet to be isolated, ascertained and thoroughly evaluated for their antidiabetic potential. Hence, in this research, our aim was to evaluate an in vitro antidiabetic potential of the *T. indica* stems ethanol extract and isolated compounds with respect to find out safe and efficacious antidiabetic agents.

## Methods

### Instruments

Melting points were recorded on a STUART SCIENTIFIC SMP10 instrument. The UV spectra were recorded on a Double beam UV-Visible Spectrophotometer 1700 (SHIMADZU Japan). ^1^H- and ^13^C–NMR spectra were recorded on an AVANCE III Bruker Spectrometer at 600 and 150 MHz, respectively. TMS was used as internal standard. ESI-MS spectra were taken on Bruker microTOF-Q spectrometer. All solvents from the extracts were evaporated to dryness using a BUCHI rotary evaporator R-200, water bath and freeze dryer (ALPHA 1–4 LD-2). The dried plant material was pulverised using Fritsch Universal Cutting Mill-Pulverisette 19-Germany. TECAN micro detection microplate reader (M 200) was used to measure the absorbance. Perkin Elmer Multi label HTS reader was used for detecting fluorescent absorbance of 2-NBDG. Evos Microscope and Dino Eye were used for taking pictures of the cells.

### Collection and preparation of plant material

Fresh stems (10 kg) of *T. indica* were collected from the local garden Taman Pertanian, Indera Mahkota, 25,200 Kuantan, Pahang DM, Malaysia. Identification of the plant was performed by the taxonomists of Taman Pertanian and Kulliyyah of Pharmacy, IIUM. Afterward, the sample was deposited in the herbarium of Kulliyyah of Pharmacy to obtain voucher specimen number (NMPC-QSTI-39) for the future references. The same plant material was compared with the already deposited specimen of the same plant.

Ten kg well cleaned fresh stems were dried in a laboratory dryer within a temperature range (30 to 40 °C) and were pulverized to a crude powdered form by applying Fritsch Universal Cutting Mill PULVERISETTE 19 (Germany) and the grinded material was made ready for the extraction process 4.7 kg (47%) to prepare ethanol extract as well as to isolate bioactive compounds [12, 13].

### Preparation of ethanol extract, fractionation and isolation of bioactive compounds

The stems powder (4.7 kg) was initially defatted using petroleum ether (b.p. 40–60 °C) and then extracted by soaking in 95% ethanol in a round bottom flask for 24 h at room temperature, filtered through Buchner funnel and finally concentrated in a reduced pressure using Buchi rotary evaporator. Recovered ethanol was again poured into the already extracted powdered material and then refluxed on the water bath for another 2–3 h. This process was repeated about 4 times till the plant material stopped giving colour to ensure maximum yield of ethanol soluble compounds from the powdered stems. The ethanol extract was eventually freeze-dried giving a final yield of 295.5 g EtOH extract (6.28%).

Fractionation of the ethanol extract was done using separatory funnel. Typically, dried ethanol extract (295.5 g) was dissolved in distilled water and treated with hexane until the hexane portion became clearly separated or visible. Collected hexane portion was recovered through rotary evaporator. The combined hexane fraction (11.2 g) was considered as non-polar extract of the stems of *T. indica*. Subsequently, the remaining hexane insoluble portion was treated with ethyl acetate (EtOAc) in the same manner. After concentrating through rotary evaporator, the collected fraction was considered as EtOAc fraction (62.28 g) of *T. indica* stems ethanol extract.

Initially, isolation of biologically active compounds was carried out by silica gel 60 (63–200 μm) column chromatography followed by small preparative column chromatographies containing silica gel 60 (63–200 μm) and sephadex LH 20. All fractions were subjected to repeated column chromatographies to isolate antidiabetic agents of *T. indica* stems in pure form [13].

### In vitro antidiabetic activity

#### Cell culture materials

Mouse 3T3-L1 fibroblast (CL-173) was obtained from the American Type Culture Collection (ATCC), Virginia, USA. Dulbecco’s-modified Eagle medium (DMEM) (with glucose and without glucose), Tryple Express (trypsin) and human recombinant insulin (4 mg/mL) were purchased from GIBCO, India. 4,5-dimethylthiazol-2-yl-2,5-diphenyltetrazolium bromide (MTT) was bought from Ameresco, Solon, Ohio 44,139, USA. Rosiglitazone, Dimethyl Sulfoxide (DMSO) and Oil Red O staining were purchased from Sigma Aldrich, USA. 2-[N-(7-Nitrobenz-2-oxa-1,3-diazol-4-yl)amino]-2-deoxy-d-glucose (2-NBDG) was obtained from Molecular Probes by Life Technologies, USA. Black microplates were collected from SPL Life Science Limited, Korea and 96 well flat bottom sterile microplates were bought from Greiner Bio-one Cellstar. Phosphate Buffered Saline (PBS) was purchased from GIBCO by Life Technologies, Invitrogen, USA. 3-isobutyl-1-methylxanthine (IBMX) was purchased from EMD Millipore Corp., USA. Dexamethasone was bought from Calbiochem, EMD Chemicals, Inc. San Diego, CA.

#### Cell culture

3T3-L1 mouse pre-adipocyte cells were cultured according to the protocol provided by the ATCC, Rockville, MD, USA. The growth media was used as DMEM containing 4.5 g/L D-glucose and L-Glutamine with no sodium pyruvate and it was completed by adding 1% penicillin-streptomycin and 5% Fetal Bovine Serum (FBS). The cells were cultured in Greiner-bio CellStar flask of 25 and 75 cm^2^. The cells were incubated in humidified atmosphere with 5% CO_2_ supply at 37 °C. The media was changed after every two days and the cells were subcultured upon 80% confluence.

#### Cell viability assay

The viability assay was carried out to determine any possible adverse effects of extract, fractions and isolated compounds on the 3T3-L1 mouse pre-adipocyte cells. Cell viability was evaluated through 3-(4,5-dimethylthiazol-2-yl)-2,5-diphenyltetrazolium bromide (MTT) assay. Maximum non-toxic concentration for each extract, fractions and isolated compounds in every bioassay was selected to maximize the potential biological activity while minimizing the potential toxic effects. The maximum non-toxic concentration was determined by treating 3T3-L1 pre-adipocyte cells with extract concentrations ranging from 0.78 to 100 μg/mL for 48 h and assessing cell percent viability. 3T3-L1 pre-adipocyte cells (2 × 10^5^ cells) were seeded in 96-well plates and allowed to get confluent for 48 h. Subsequently, they were treated with extract, fractions and isolated compounds (dose: 0.78–100 μg/ml) for 48 h. Later, the cells were treated with MTT at 5 mg/ml. Every well was treated with 20 μL of MTT and incubated for 4 h. Finally, 100 μL of DMSO was transferred to every well to dissolve the water-insoluble purple formazan crystals [14]. Plates were kept at normal temperature wrapped in the aluminum foil. Absorbance values were measured by TECAN micro detection microplate reader (M 200) at 570 nm.

Cell percent viability (%) = [(mean A sample- mean A blank) / (mean A control-mean blank)] X 100.

### Adipogenesis

#### Induction of Adipogenesis

Insulin is a potent adipogenic hormone that triggers an induction of a series of transcription factors governing differentiation of pre-adipocytes into mature adipocytes. The transformation of the pre-adipocytes to mature adipocytes is known as adipogenesis or differentiation. The adipogenesis was induced according to the protocol reported previously [12, 13, 15, 16]. Briefly, the pre-adipocytes were seeded in 96 well flat bottom sterile microplates. Later, the same cells were treated with the adipogenic cocktail after two days upon 90% confluence. The day was considered as day zero. At the end of the adipogenesis period, more than 90% of the cells contained lipid droplets that could be viewed under low power magnification. This protocol [15] was slightly altered for the adipogenesis experiment, whereby adipogenesis was initiated 2 days post-confluence and total adipogenesis time was 5 to 6 days instead of 6–8 days. The adipogenic cocktail (differentiation media, DM) was modified by insulin, compound/extract and rosiglitazone. Adipogenesis of fibroblasts into mature adipocytes was confirmed by Oil Red O staining (Table 1).

**Table 1:** Adipogenic cocktail

Group	IBMX	Dexamethasone	Insulin	Rosiglitazone	Compounds
Negative control	------	------------	--------	--------	--------
Control	0.5 mM	1 μM	10 μg/ml	--------	---------
Positive control	0.5 mM	1 μM	10 μg/ml	10 μM	--------
Insulin like activity	0.5 mM	1 μM	---------	--------	Three different concentrations
Insulin-sensitizing activity	0.5 mM	1 μM	10 μg/ml	-------	Three different concentrations

Three different concentrations of wogonin (12.5, 25 and 50 μg/ml), norwogonin (25, 50 and 100 μg/ml) and techtochrysin (12.5, 25 and 50 μg/ml) were determined according to the safety of the cells initially confirmed through MTT assay. The 3T3-L1 pre-adipocytes were incubated for 2 days in the differentiation media. On ‘day 2’, the media was changed to insulin media (IM) (viz. 10 μg/mL insulin in DMEM) for 2 days. Finally, on day 4, the DMEM was applied and was changed in every 2 days for 6–8 days.

#### Oil red O staining

Oil Red O (Sudan Red 5B, C_26_H_24_N_4_O) is a lysochrome (fat-soluble dye) diazo dye generally used to stain lipids and neutral triglycerides on frozen sections. It is red powder with maximum absorption observed at 518 nm. After adipogenesis, the cells were fixed with 10% formalin in phosphate-buffered saline (PBS) for 1 h at room temperature. Afterwards, the cells were washed 3 times with PBS and stained with freshly prepared Oil Red O (3 parts of Oil Red O of 0.6% in 2 parts of deionized water) from the stock solution for 1 h. Cells were again washed with distilled water and approximately 1 mL isopropanol was further added. Oil Red O staining was extracted by isopropanol after 5 min. Finally, the absorbance was measured using microplate reader at 520 nm [15].

### 2-NBDG uptake in 3T3-L1 adipocyte cells

2-NBDG (2-(*N*-(7-Nitrobenz-2-oxa-1,3-diazol-4-yl)Amino)-2-Deoxyglucose, C_12_H_14_N_4_O_8_) is a fluorescent glucose analog which is used to screen glucose uptake in live cells, as an indicator of cell viability. 2-NBDG typically displays excitation/emission maxima of ~465/540 nm and can be visualized using optical filters designed for fluorescein. To determine whether ethanol extract, fractions and isolated compounds could exert insulin-like or insulin-sensitizing effects on glucose uptake, 3T3-L1 adipocytes were treated with the maximum non-toxic concentrations of ethanol extract, sub-fractions and isolated compounds to stimulate 2-NBDG in the absence and presence of insulin and rosiglitazone (insulin-sensitizer). The 2-NBDG uptake test was carried out by following the protocol described by Alonso-Castro et al. with some modifications [17]. Briefly, the pre-adipocytes were treated with the adipogenic cocktail to differentiate into mature adipocytes using the same protocol used for adipogenesis control group. When the adipogenesis had been completed on day 8, the adipocytes were incubated in DMEM (serum and glucose free) for two days. Afterwards, the serum and glucose starving adipocytes were treated with 80 μM 2-NBDG and sample (in different concentrations) with insulin (10 μg/mL) or with rosiglitazone (10 μM) for 48 h. At the same time, sample was seeded with insulin to investigate the possible insulin-sensitizing effect. The cultures were washed with PBS to get rid of free 2-NBDG upon finishing the incubation. Finally, the fluorescence retained in the cell monolayer was measured by fluorescence microplate reader (Perkin Elmer Multi label HTS reader) at 485 nm (excitation wavelength) and 535 nm (emission wavelength), respectively. The 100% specific 2-NBDG uptake was determined by subtracting control having 2-NBDG and DMEM from control having 2-NBDG and insulin (Table 2).

**Table 2:** 2-NBDG uptake groups

Groups	Cells	Insulin	2-NBDG	Rosiglitazone	Compounds
Negative control	3T3-L1 preadipocytes	---------	80 μM	---------	---------
Control	3T3-L1 adipocytes	10 μg/ml	80 μM	---------	---------
Positive control	3T3-L1 adipocytes	10 μg/ml	80 μM	10 μM	------------
Insulin-like activity	3T3-L1 adipocytes	---------	80 μM	---------	Three different concentrations
Insulin-sensitizing activity	3T3-L1 adipocytes	10 μg/ml	80 μM	10 μM	Three different concentrations

100% specific absorbance = (Absorbance of insulin induced 2-NBDG uptake - Absorbance of non-insulin induced 2-NBDG uptake).

### Statistical analysis

Data were interpreted using IBM SPSS (version 20) and a minimum of three (*n* = 3) replicates were performed for each data on different days. Graphical representations of all data were done by Microsoft Excel. The analysis was done by one-way ANOVA post hoc and followed by dunnett multiple comparison test. The untreated control was taken as dependent variable and rest of the groups were compared with it. A *p < 0.05 was considered statistically significant and **p < 0.005 was considered highly significant.

## Results

**Fig. 1:**
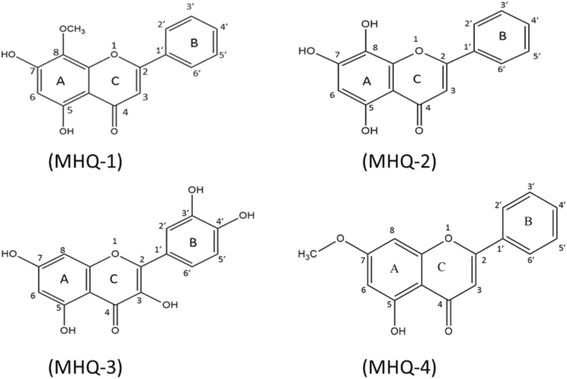
Chemical structure of wogonin (MHQ-1), norwogonin (MHQ-2), quercetin (MHQ-3) and techtochrysin (MHQ-4)

The in vitro antidiabetic activity of the isolated monoflavonoids from *T. indica* stems ethanol extract as well as their safe nature (non-toxic behavior evaluated through MTT assay on adipocytes) using in vitro diabetic model are being reported for the first time through this research study Fig. 1 depicts the flow chart of the current study.

### Isolation and structure characterization of bioactive compounds

**Fig. 2:**
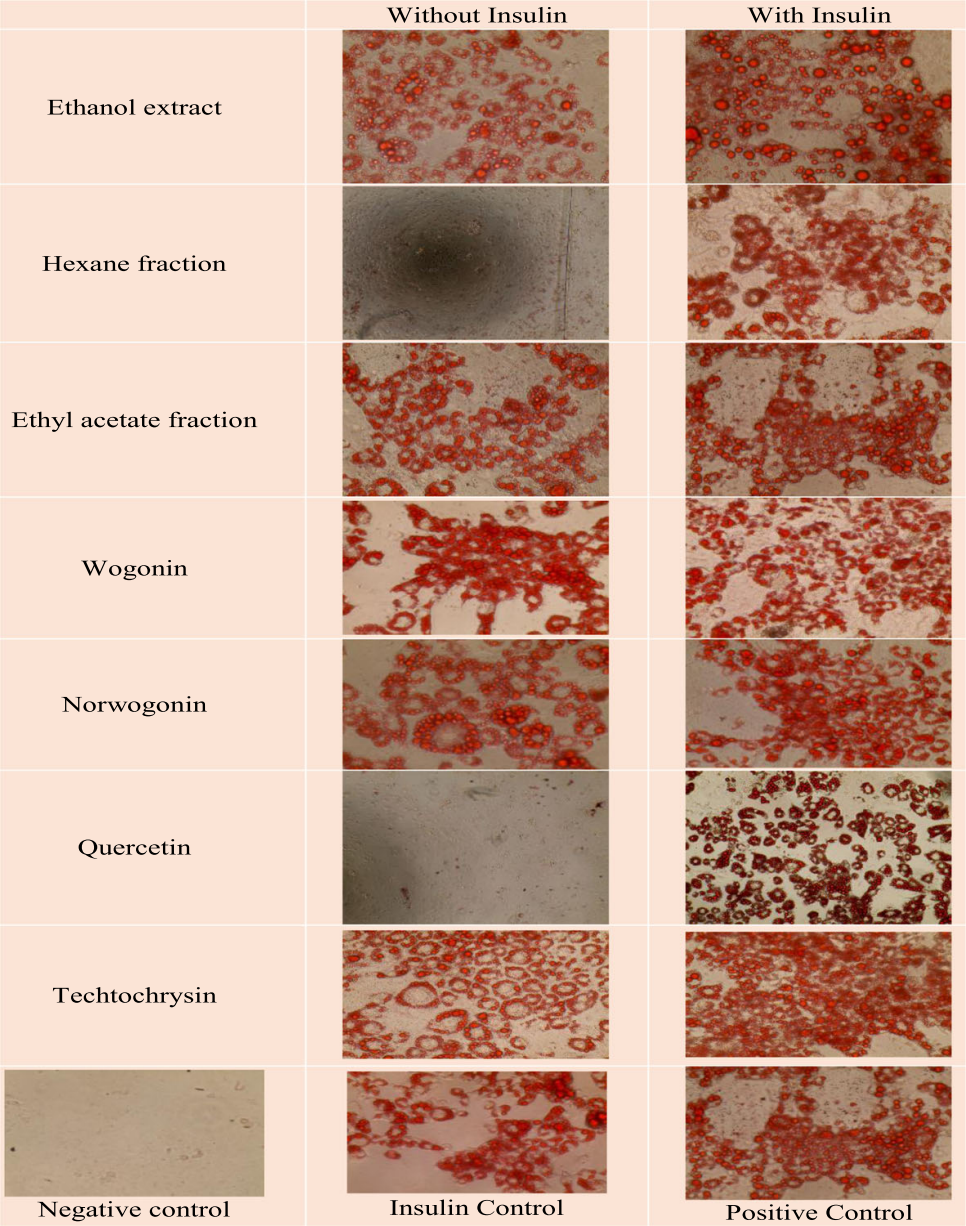
Oil-red-O staining of 3T3-L1 adipocytes on day 10. Results showed to induce differentiation at indicated concentrations in the presence or absence of insulin (10 μg/mL). Cultures in basal medium and insulin served as positive control. Cells treated with rosiglitazone served as drug control

Active fraction (ethyl acetate fraction) of *T. indica* stems ethanol extract using repeated silica gel and sephadex LH 20 column chromatographies afforded four different monoflavonoids viz., MHQ-1 (wogonin), MHQ-2 (norwogonin), MHQ-3 (quercetin) and MHQ-4 (techtochrysin) (Fig. 2). These compounds were identified by spectroscopic analysis. Their spectral data were further evaluated and compared with the previously reported spectral data of the similar compounds already isolated from different plants.

MHQ-1 (Wogonin): Yellow crystal; mp: 205–206 °C; U.V_max_ nm MeOH: (MeOH only): 211.7, 276; (MeOH/NaOMe, immediately): 242, 284, 381; (MeOH/NaOMe, after ten min): 242, 284.5, 381.50; (MeOH/NaOAc): 243, 284, 381; (MeOH/NaOAc/H_3_BO_3_): 279, 351; (MeOH/AlCl_3_): 284.50, 325.1, 404.7; (MeOH/AlCl_3_/HCl): 287, 329, 403.5; (MeOH/NaOH): 242, 286.50, 380; IR (KBr) *ν*_max_ cm^−1^: 3190, 2923, 2860, 1732, 1656, 1607, 1578, 1559, 1508, 1451, 1414, 1355, 1325, 1298, 1264, 1248, 1206, 1112, 1161, 1112, 1019, 987, 842, 787, 763, 682, 671, 661, 556; ^1^H–NMR [600 MHz, Acetone-d_6_,δ (ppm)]: δ 6.835 (s, 1H, H-3), δ 6.349 (s, 1H, H-6), δ 8.136 (m, 2H, H-2′/H-6′), δ 7.653 (m, 3H, H-3′/H4′/H5′), δ 3.983 (s, OCH_3_, 3H, H-8a), 12.591 (s. 1H, OH-5); ^13^C–NMR [150 MHz, Acetone-d_6,_ δ (ppm)]: δ 157.34 (C-2), δ 105.18 (C-3), δ 182.42 (C-4), δ 157.15 (C-5), δ 99.00 (C-6), δ 163.59 (C-7), δ 127.93 (C-8), δ 149.91 (C-9), δ 104.48 (C-10), δ 131.42 (C-1′), δ 129.25 (C-2′), δ 126.37 (C-3′), δ 131.95 (C-4′), δ 126.37 (C-5′), δ 129.25 (C-6′), δ 61.07 (C-8a); ESI-MS m/z 284 [M]^+^; 283 [M-H]^−^; 285 [M + H]^+^ corresponding to (C_16_H_12_O_5_) [18].

MHQ-2 (Norwogonin): Golden yellow crystal; mp: 258–260 °C; U.V_max_ nm MeOH: (MeOH only): 276.50; (MeOH/NaOMe, immediately): 377, 282.50, 242.50; (MeOH/NaOMe, after 10 min): 377, 283, 244; (MeOH/NaOAc): 372, 283, 243; (MeOH/NaOAc/H_3_BO_3_): 279, 241; (MeOH/AlCl_3_): 291.50, 248.50; (MeOH/AlCl_3_/HCl): 291.50, 249.50; (MeOH/NaOH): 377.50, 284.50, 242.50;IR (KBr) *ν*_max_ cm^−1^: 3283.70, 3060.50, 2356.10, 1650.89, 1595.64, 1573.38, 1549.28, 1520.32, 1445.31, 1415.84, 1369.73, 1321.16, 1261.95, 1242.98, 1207.05, 1176.24, 1157.89, 1011.02, 956.88, 833.22, 773.96, 718.28, 676.83, 661.83, 566.38, 503.53, 470.78**;** ^1^H–NMR [600 MHz, MeOD-d_4_,δ (ppm)]: δ 6.783 (s, 1H, H-3), 6.365 (s, 1H, H-6), 7.635 (m, 3H, H-3′/H4′/H5′), 8.165 (m, 2H, H-2′/H-6′), 12.356 (s, 1H, OH-5); ^13^C–NMR [150 MHz, MeOD-d_4,_ δ (ppm)]: δ 153.17 (C-2), 104.98 (C-3), 184.54 (C-4), 154.54 (C-5), 98.65 (C-6), 163.62 (C-7), 131.51 (C-8), 145.67 (C-9), 104.21 (C-10), 124.88 (C-1′), 129.00 (C-2′, C-6′), 126.56 (C-3′, C-5′), 131.75 (C-4′); ESI-MS m/z 270 [M + H]^+^; 269 [M-1H]^−^ corresponding to C_15_H_10_O_5_ [19].

MHQ-3 (Quercetin): Yellow crystal, mp: 315–316 °C; U.V_max_ nm: (MeOH only): 242.50, 281, 330; (MeOH/NaOMe, immediately): 243.50, 280.50, 331.50; (MeOH/NaOMe, after 10 mins.) 246, 333; (MeOH/NaOAc): 255.50, 376.50; (MeOH/NaOAc/H_3_BO_3_): 259, 298.50, 386; (MeOH/AlCl_3_) 222, 264.50; (MeOH/AlCl_3_/HCl): 222, 266.50, 362.50; (MeOH/NaOH) 219, 255, 370; IR (KBr) *ν*_max_ cm^−1^: 3257.4, 1660.9, 1603.2, 1558.5, 1519.3, 1447.0, 1407.4, 1378.2, 1316.3, 1258.7, 1213.5, 1196.2, 1165.3, 1130.3, 1091.4, 1013.6, 840.7, 818.7, 794.6, 782.7, 720.4, 672.4, 636.9, 601.5, 488.8; ^1^H–NMR [600 MHz, Acetone-d_6_,δ (ppm)]: δ 6.260 (d, J = 2.4 Hz, 1H, H-6), 6.516 (d, J = 1.8, 1H, H-8), 6.997 (d, J = 8.4 Hz, 1H, H-5′), 7.700 (dd, J = 1.8, 8.4 Hz, 1H, H-6′), 7.819 (d, J = 2.4 Hz, 1H, H-2′), 12.45 (1H, s, OH-5); ^13^C–NMR [150 MHz, Acetone-d_6,_ δ (ppm)]: 175.60 (C-4), 164.07 (C-7), 161.18 (C-5), 156.88 (C-9), 147.36 (C-4′), 146.00 (C-2), 144.86 (C-3′), 135.81 (C-3), 122.84 (C-1′), 120.56 (C-6′), 115.33 (C-5′), 114.78 (C-2′), 103.17 (C-10), 98.17 (C-6), 93.56 (C-8); ESI-MS m/z 301 [M-2H]^−^, 303 [M + H]^+^ corresponding to C_15_H_10_O_7_ [20].

MHQ-4 (Techtochrysin) (5-hydroxy-7-methoxyflavone): Light greenish yellow crystal; mp: 295 °C; U.V_max_ nm MeOH: (MeOH only): 237, 271, 354; (MeOH: NaOMe, immediately): 351.50; (MeOH/NaOMe, after ten min): 357.50; (MeOH/NaOAc): 236.50, 266.50, 349.50; (MeOH/NaOAc/H_3_BO_3_): 260.50, 317; (MeOH/AlCl_3_): 252, 310.50; (MeOH/AlCl_3_/HCl): 264; (MeOH/NaOH): 255.50, 314; IR (KBr) *ν*_max_ cm^−1^: 3093.0, 1633.2, 1582.0, 1569.1, 1510.7, 1494.6, 1465.4, 1432.5, 1395.7, 1310.5, 1264.4, 1243.0, 1220.0, 1166.1, 1116.9, 1101.4, 1049.9, 993.0, 960.0, 909.3, 843.6, 797.3, 759.7, 685.7, 670.7, 670.7, 537.2, 498.6; ^1^H–NMR [600 MHz, MeOD-d_4_, δ (ppm)]: δ 6.68 (s, 1H, H-3), 6.45 (d, J = 2.4 Hz, 1H, H-6), 6.60 (d, J = 2.4, 1H, H-8), 7.97 (dd, J = 1.8,4.2 Hz, 2H, H-2′/H-6′), 7.58 (m, 3H, H-3′/H-4′/H5′), 3.90 (s, 3H, 7-OCH_3_); ^13^C–NMR [150 MHz, MeOD-d_4,_ δ (ppm)]: 162.72, 108.73, 180.23, 163.32, 97.87, 165.57, 96.66, 161.58, 108.50, 132.68, 130.37, 127.35, 132.88, 127.35, 130.37, 56.64; ESI-MS m/z 268 [M + H]^+^, 267 [M-1H]^−^ corresponding to C_16_H_12_O_4_ [21].

### In vitro bioactivity determination

#### MTT assay

MTT viability assay was performed on 3T3-L1 pre-adipocytes to assess the safety of the plant’s ethanol extract, sub-fractions and isolated compounds. In this study, the IC_50_ (concentration at which sample kills 50% of the cells) was considered the safe concentration (viz. isolated compounds, extracts and fractions) for the adipocyte cells in order to assess antidiabetic evaluation of test substances. The concentrations at which more than 50% cells found to be alive were selected for an in vitro antidiabetic evaluation. Eight concentrations of each sample (0.78 to 100 μg/mL) had been used to check whether it were safe to the adipocytes cells or not. Results suggested that the *T. indica* stems ethanol extract inhibited 18.60% cells at its highest concentration (100 μg/mL) which was found to be highly significant (**p < 0.005) in comparison to the control group. So, the ethanol extract was considered safe for the cells at its highest concentration (viz. 100 μg/mL). Similarly, both fractions (hexane and ethyl acetate) were also found to be safe at their highest concentrations viz. 35.27% and 21.40%, respectively, and both varied from the control significantly (**p < 0.005 inhibition) (Table 3).

**Table 3:** MTT viability assay results

Sample	Dose	% Inhibition
Ethanol (95%) extract	100 μg/mL	18.60
Hexane fraction	100 μg/mL	35.27
Ethyl acetate fraction	100 μg/mL	21.39
MHQ-1 (Wogonin)	25 μg/mL	51.96
MHQ-2 (Norwogonin)	100 μg/mL	22.64
MHQ-3 (Quercetin)	100 μg/mL	25.48
MHQ-4 (Techtochrysin)	50 μg/mL	49.39

Phenolic compounds isolated from the ethyl acetate fraction of *T. indica* stems ethanol extract viz. wogonin (MHQ-1) showed IC_50_ at 25 μg/mL (51.97% inhibition) and techtochrysin (MHQ-4) showed IC_50_ at 50 μg/mL (49.40% inhibition). However, the remaining two phenolic compounds (norwogonin and quercetin) were found to be safe up to 100 μg/mL. The percent inhibitions at their highest concentrations of norwogonin (MHQ-2) and quercetin (MHQ-3) were found to be 22.75% and 25.48%, respectively. So, it was concluded that these compounds up to 100 μg/mL can be safely used to evaluate bioactivity due to their safe profile at the tested concentration through MTT assay.

#### Adipogenesis

**Fig. 3:**
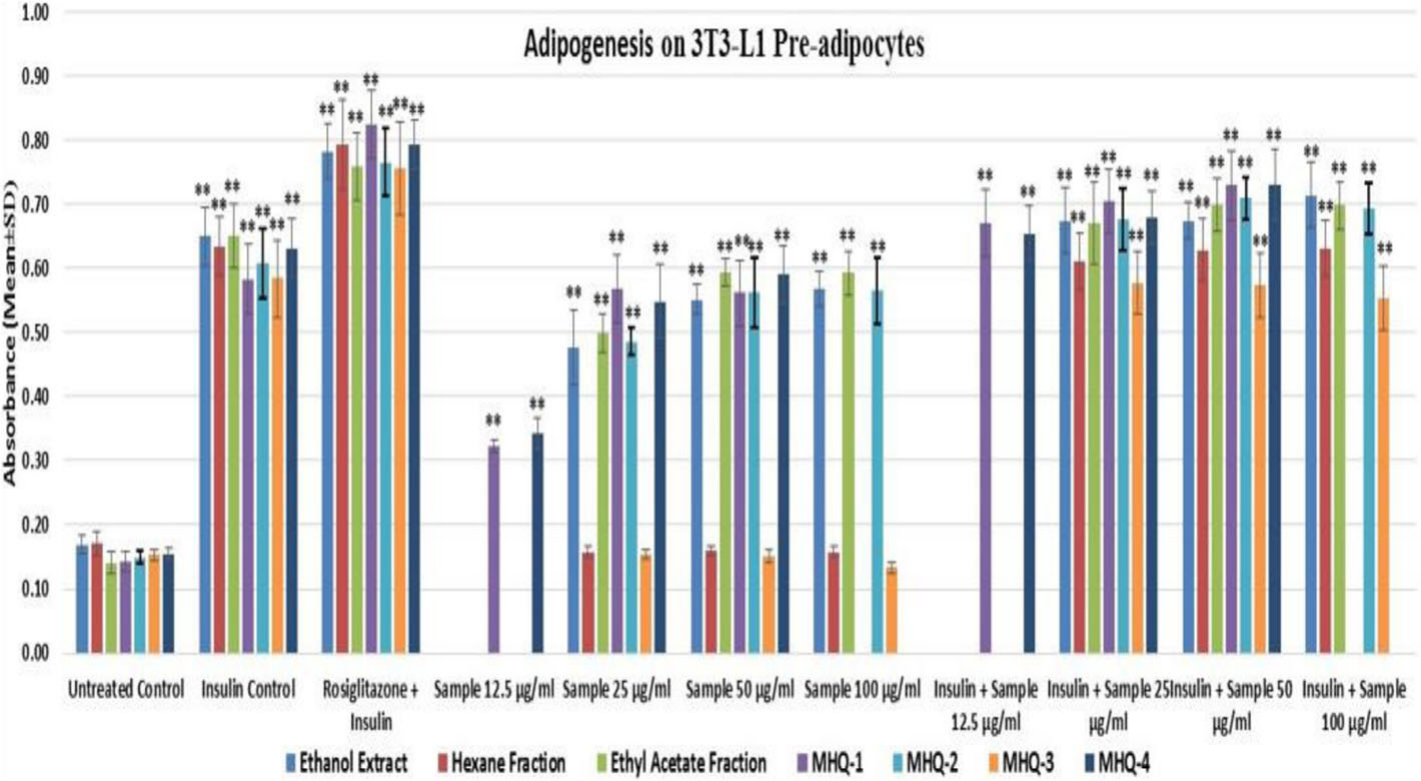
*T. indica* stems ethanol extract, two fractions (hexane and ethyl acetate) and isolated four phenolic compounds (MHQ-1, MHQ-2, MHQ-3, MHQ-4) were investigated for their ability to enhance adipogenesis in the absence and presence of insulin (10 μg/mL) and rosigiltazone (10 μM). The results showed in absorbance (Mean ± SD), *n* = 3 per group and triplicate of each group

**Fig. 4:**
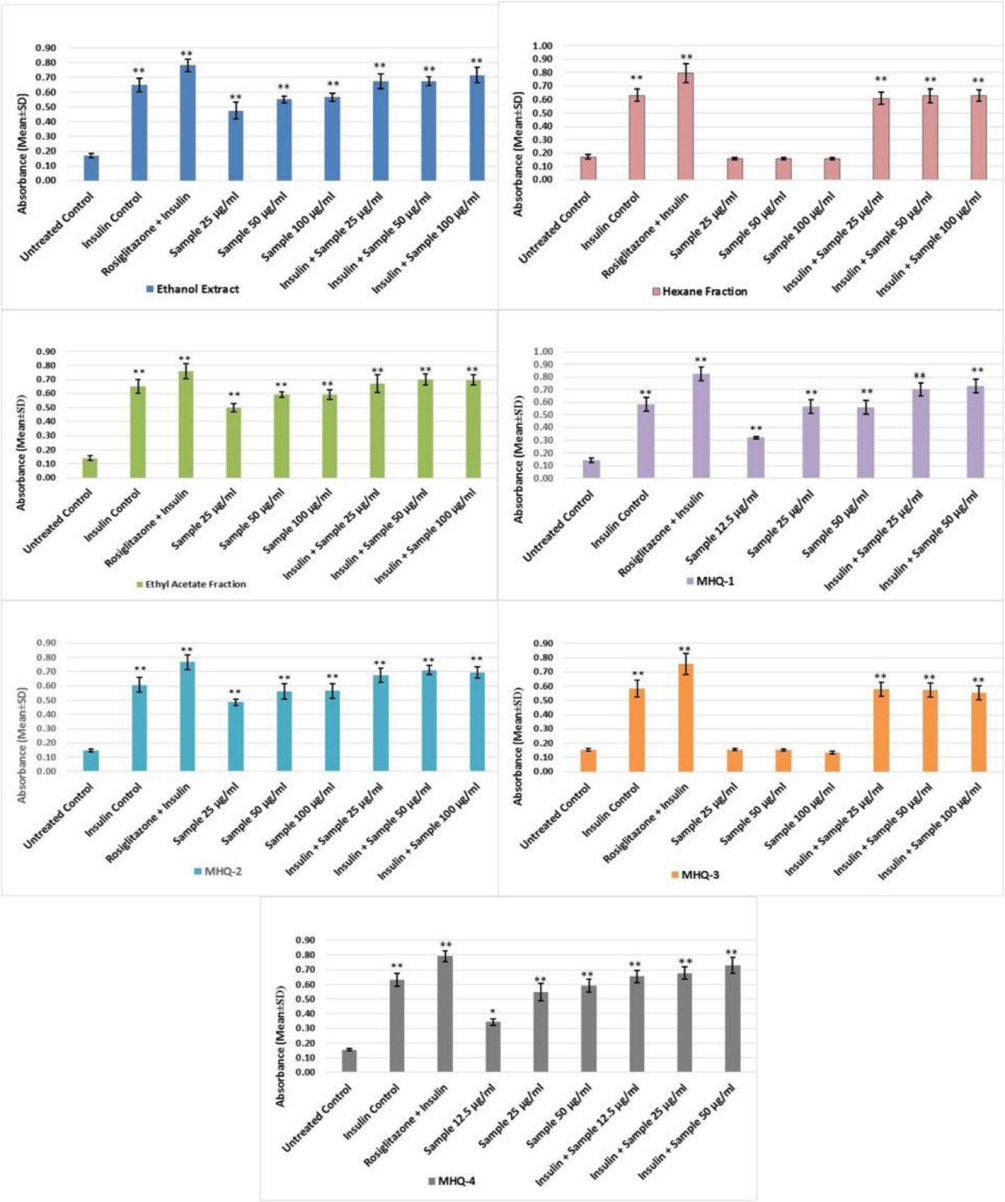
*T. indica* stems ethanol extract, two fractions (hexane and ethyl acetate) and four phenolic compounds (MHQ-1, MHQ-2, MHQ-3, MHQ-4) were investigated for their ability to enhance adipogenesis in the absence and presence of insulin (10 μg/mL) and rosigiltazone (10 μM) at three different safe concentrations obtained according to MTT viability assay (12.5–50 μg/mL). Cells in DMEM without any treatment were considered as untreated control. Cells treated with insulin and rosiglitazone were taken as insulin control and positive control. Data expressed in percentage (rosiglitazone was taken as 100%) mean ± SD, *n* = 9 (biological triplicate each containing *n* = 3). One-way ANOVA showed significant value, **p < 0.005

**Fig. 5:**
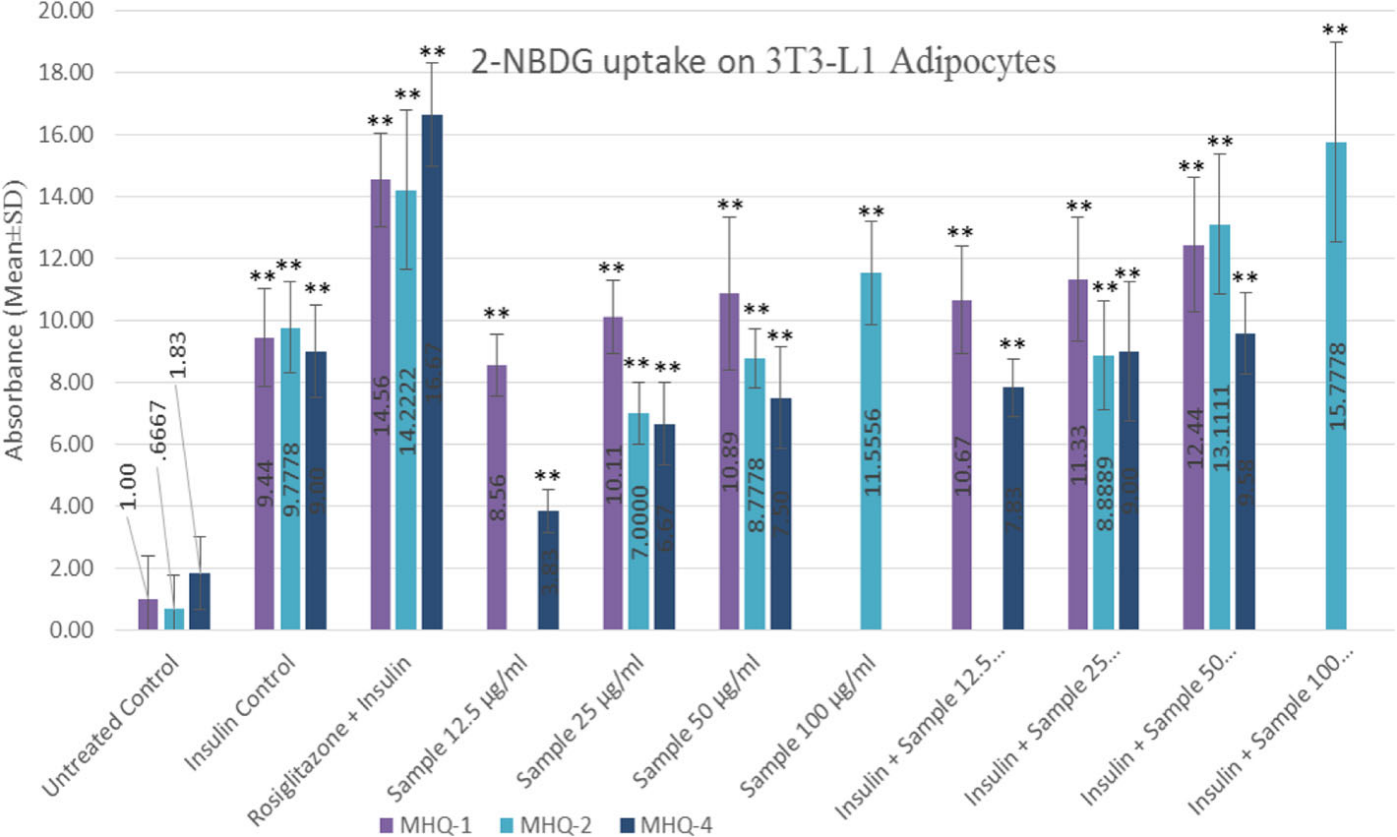
Wogonin (MHQ-1), norwogonin (MHQ-2) and techtochrysin (MHQ-4) were evaluated to stimulate 2-NBDG uptake in 3T3-L1 adipocytes in the absence and presence of insulin at indicated concentration 12.5–100 μg/mL, respectively. Cultures in basal medium and insulin served as control. Cells treated with rosiglitazone were considered as positive control which represents insulin-sensitizing activity. Data expressed in mean ± SD, *n* = 9 (Three biological triplicate each containing minimum *n* = 3). One-way ANOVA showed significant value, **p < 0.005

In this experiment, ethanol extract of the stems of *T. indica’s* stems ethanol extract, its sub-fractions and isolated phenolic compounds from the bioactive fraction were evaluated in order to check whether they can induce adipogenesis in the presence and absence of insulin. Rosiglitazone was taken as positive control which was technically used as a marker for insulin-sensitizing activity of the samples. The results showed that the *T. indica* stems ethanol (95%) extract, ethyl acetate fraction and three of the four phenolic compounds (MHQ-1, MHQ-2 & MHQ-4) showed effects in a dose-dependent manner similar to insulin- like activity and also insulin sensitizing activity. However, non-polar fraction (hexane fraction) and quercetin (MHQ-3) showed no effect on stimulating adipogenesis without insulin. Moreover, both did not exert any insulin sensitizing effect in comparison to rosiglitazone (Figs. 3, 4 and 5).

#### Fluorescence glucose uptake

**Fig. 6:**
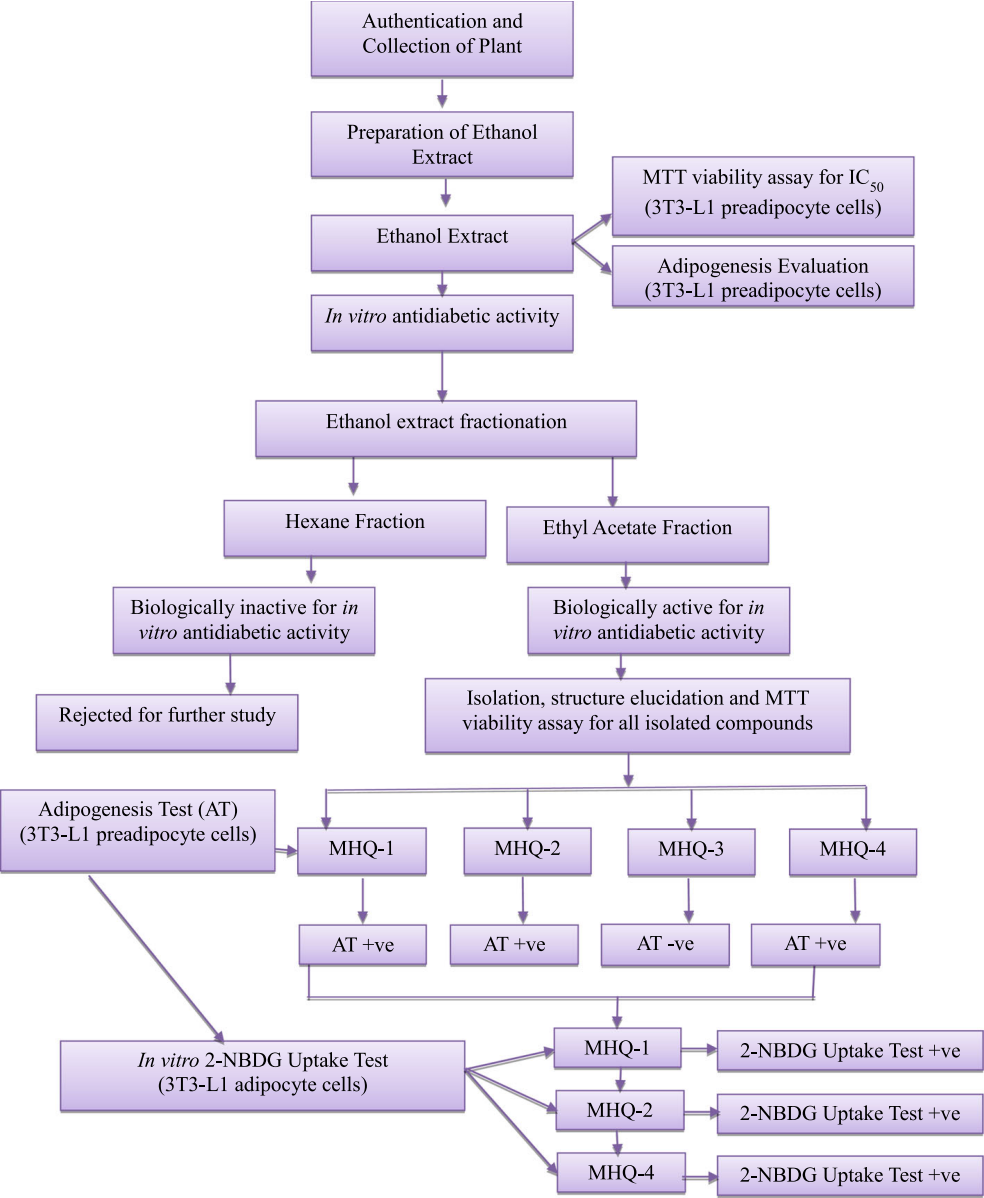
Flow chart of the study

This experiment was designed to evaluate the insulin-like and insulin sensitizing activity for the isolated phenolic compounds. Hence, wogonin, norwogonin and techtochrysin were evaluated on matured adipocytes to stimulate 2-NBDG in the absence and presence of insulin and rosiglitazone (insulin-sensitizer). The results in our study show an increase stimulation of the 2-NBDG uptake in the absence of insulin. It was found out that all three phenolic compounds exerted a dose-dependent effect as the activity was found to increase with the increased concentrations of all three phenolic compounds. Interestingly, it was also found out that 2-NBDG uptake by wogonin was higher than the insulin (control) at its highest concentration i.e. 50 μg/mL. Norwogonin also exhibited 2-NBDG uptake activity higher than the insulin by showing higher fluorescence absorbance at its highest concentration i.e. 100 μg/mL. In the same experiment, rosiglitazone was taken as a positive control to determine insulin-sensitizing effect on 3T3-L1 adipocytes. The results suggested that all three phenolic compounds exert insulin sensitizing effect in a dose-dependent manner. Fluorescence absorbance of the wogonin was slightly less than the rosiglitazone at its highest concentration i.e. 50 μg/mL, however, norwogonin showed more 2-NBDG uptake than the rosiglitazone at its highest concentration i.e. 100 μg/mL. Moreover, techtochrysin also showed 2-NBDG uptake on matured adipocytes, which was, however, less than the wogonin and norwogonin at its highest concentration i.e. 50 μg/mL. Techtochrysin exhibited concentration-dependent 2-NBDG uptake by showing a slightly higher absorbance (0.58) than the insulin at its highest concentration in fluorescence microplate reader. However, techtochrysin did not display any significant insulin sensitizing activity when it was compared with the absorbance of the rosiglitazone (Fig. 6).

## Discussion

Adipocytes are the major site of insulin action and, thereby, play an important role in glucose metabolism as well as in the regulation of whole-body glucose homeostasis. The widespread epidemics of obesity and type-2 diabetes mellitus (T2DM) suggest that both conditions are closely interconnected. The increased incidence of extreme visceral obesity and obesity-related cardiovascular risk factors are closely related to the rising prevalence of cardiovascular diseases and T2DM [12, 13]. The prevalence of obesity has become a chief public health apprehension worldwide. Obesity is described by the accretion of overabundance fat in adipose tissues [22] and increased deposition of cytoplasmic triglycerides, which might lead to various metabolic and chronic ailments such as cardiovascular diseases, T2DM, cancer etc. [23]. The development of obesity in adults is also accompanied by substantial differentiation of preadipocytes into adipocytes [15]. Recently, the research studies on adipocytes have been growing enormously. Consequently, adipocytes are evolving as a major drug target for diabetes and obesity-mediated metabolic syndromes [24]. Adipose tissues are not only recognized for their capacity to store the excess of dietary energy in the form of triglyceride [25], however, they are also known to play a vital role in the regulation of energy metabolism [26, 27]. During the process of adipogenesis, the pre-adipocytes undergo growth arrest and they are differentiated to mature adipocytes. There is a dramatic increase in the adipocyte gene expression during this process which includes adipocyte fatty acid binding proteins, lipid metabolizing enzymes etc. A number of transcription factors have been reported to be engaged in the complex adipogenesis process where glucose uptake and glycolysis pathway are considered noteworthy [28]. These transcription factors include peroxisome proliferator-activated receptor-γ (PPARγ), glucose transporter-4 (GLUT4), and adipokines such as leptin. One of the most important processes of adipogenesis is PPAR-γ activation by the ligands. PPARγ is predominantly expressed in adipose tissues and plays a central role in adipose tissue functions [29]. Moreover, PPAR-γ is responsible for the genes linked to insulin signaling, glucose and lipid metabolism in mature adipocytes [30]. Reduced expression of PPAR훾 has been shown to be effective in inhibiting the adipogenesis of 3 T3- L1 cells [31]. Glucose homeostasis in the body is mainly mediated by translocation of the insulin-responsive glucose transporter GLUT4. An impaired GLUT4 translocation plays a crucial role in the pathogenesis of insulin resistance as one of the earliest events during the development of T2DM. GLUT4 is a high affinity glucose transporter predominantly expressed in insulin-sensitive tissues such as muscle and adipocytes [32]. Increased expression and plasma membrane translocation of GLUT4 have been found to lower blood glucose, enhance glucose transport and utilization [33]. Leptin is produced mainly by adipocytes and also found in low levels in the gastric fundic epithelium, intestine, skeletal muscle, mammary epithelium, placenta, and brain [34]. High levels of circulating leptin in adipose tissues characterize human obesity [35] and increased levels of body fat [36].

3T3-L1 pre-adipocytes after undergoing the differentiation to adipocytes serve as excellent in vitro models and are considered valuable tools in understanding the glucose metabolism. In recent research studies, 3T3-L1 pre-adipocytes have been extensively used for the adipogenesis. The pre-adipocytes are adherent fibroblast-like in shape and they form confluent monolayer. Pre-adipocytes are differentiated into adipocytes through an inducer known as adipogenic cocktail which includes insulin, phosphodiesterase inhibitor methylisobutylxanthine (IBMX), fetal bovine serum (FBS) and the synthetic glucocorticoid dexamethasone [37]. Our study was designed to evaluate the activity by altering the adipogenic cocktail. The insulin like activity of the compounds was determined by replacing the insulin from the adipogenic cocktail and the insulin-sensitizing activity was determined in the presence of insulin to evaluate whether it can sensitize or increase the effect of insulin. In our study, we used *T. indica* stems ethanol extract, its sub-fractions and isolated compounds from the bioactive fraction in order to check whether they can induce adipogenesis in the presence or absence of insulin. Rosiglitazone, a thiazolidinediones drug, was taken as positive control which is an insulin-sensitizing agent that acts by improving the sensitivity of peripheral tissues to insulin through activating PPAR-γ receptor [38, 39]. The results showed that the *T. indica* stems ethanol extract, ethyl acetate fraction and three monoflavonoids isolated from the ethyl acetate fraction viz. wogonin, norwogonin and techtochrysin showed effects in a dose dependent manner similar to insulin-like- and insulin sensitizing activities. However, non-polar fraction (hexane fraction) showed no effect on stimulating adipogenesis in the absence of insulin. Moreover, the hexane fraction also did not show any insulin sensitizing effect in comparison to rosiglitazone. Stems ethanol extract, ethyl acetate fraction and three monoflavonoids (wogonin, norwogonin and techtochrysin) at their highest concentration showed absorbance almost similar to control (insulin) and also exhibited similar activity like rosiglitazone.

PPARγ ligands can affect the adipocyte differentiation and are reported to have an effect on glucose uptake in 3T3-L1 adipocytes [39]. Hence, the next objective of this research study was an attempt to assess the effect of isolated compounds on the glucose uptake and insulin sensitizing effects. In adipocytes, basal (cells treated with normal glucose without the presence of insulin and 2-NBDG) and insulin-stimulated glucose uptake activities require a glucose transporter. Insulin can accelerate glucose entry by affecting the translocation of GLUT4 from intracellular stores to the plasma membrane [40]. In general, it is known that GLUT4 provides insulin-stimulated glucose transport in adipocytes [41]. Furthermore, glucose uptake in adipocytes is the consequences of stimulation of insulin receptors by insulin. This process consists of translocation of GLUT4 [42]. During adipogenesis, the expression of GLUT4 is increased and in the presence of insulin, the GLUT4 is translocated to plasma membrane. Subsequently, the glucose uptake by the cells is increased [43]. Moreover, GLUT4 is expressed only in the adipocytes and its expression is regulated by PPARγ. GLUT4, which transports glucose from blood into tissue, is the principal glucose transporter among several isotypes of glucose transporters in insulin-sensitive tissues such as skeletal muscle and adipocytes [44]. Decrease in the translocation of GLUT4 to the plasma membrane has been found to be the principal cause of insulin resistance [45], and therefore, it is required to activate GLUT4 in the skeletal muscle to improve insulin resistance and to maintain blood glucose homeostasis. Metformin, is one of the most commonly prescribed antidiabetic drugs worldwide, can enhance the insulin-stimulated glucose uptake by increasing the GLUT4 content at the cell surface [46]. Rosiglitazone belongs to thiazolidinediones, also known as “glitazones,” binds to peroxisome proliferator-activated receptors (PPARs), a type of nuclear regulatory proteins involved in transcription of numerous genes regulating glucose and fat metabolisms. They act as “insulin sensitizers” without increasing insulin secretion. Thiazolidinediones do not increase insulin release like the sulfonylureas but increase the response to insulin. Our experiments were designed to evaluate insulin-like and insulin sensitizing activities. Flavonoids are a group of polyphenolic compounds which are known to exert a variety of biological effects including antidiabetic effect. Hence, we evaluated all the three monoflavonoids (wogonin, norwogonin and techtochrysin) on the matured adipocytes to stimulate 2-NBDG in the absence and presence of insulin and rosiglitazone (insulin-sensitizer).

MTT viability test helps to determine the safe concentration of the tested compounds used in the bioactivity experiments on pre-adipocytes. Hence, in our research study, a total of 8 different concentrations (doses) were initially evaluated and finally 3 safe doses were selected to evaluate their bioactivities in the experiments. All groups were compared with the untreated control group to check the significant differences. It was observed that the insulin control and rosiglitazone control groups were significantly different from the untreated control group. As the mechanism of action for insulin sensitizer is to increase the insulin activity, we treated rosiglitazone along with insulin to observe the insulin-sensitizing activity. Moreover, the treatment groups without insulin were compared with the insulin groups to understand the insulin-like activity of the groups whereas the groups containing the insulin and sample (compounds/extracts) were compared to rosiglitazone group to check the insulin-sensitizing activity. It was observed that the effect of adipogenesis was manifested in a dose dependent manner. The ethanol extract, ethyl acetate fraction, wogonin, norwogonin and techtochrysin showed insulin like activity. Moreover, ethanol extract, ethyl acetate fraction, wogonin, norwogonin and techtochrysin exhibited activity similar to insulin at their highest dose. The groups also showed same trend when they were compared with rosiglitazone-insulin combination. In contrast, however, the hexane fraction and quercetin showed no significant adipogenesis when they were evaluated without insulin. Additionally, no insulin sensitizing activity was observed with the hexane fraction and quercetin either.

Our study showed an increase stimulation of the 2-NBDG uptake in the absence of insulin. We can see a dose-dependent increase for all the three phenolic compounds. All three compounds (i.e. wogonin, norwogonin and techtochrysin) exerted a dose-dependent activity as the activity was found to increase with the increased concentration of all compounds. Interestingly, it was also observed that, 2-NBDG uptake by wogonin was higher than the insulin control at its highest concentration (50 μg/mL). Same results were manifested by the norwogonin as well. The fluorescence absorbance at the highest concentration i.e., 100 μg/mL was found to be much higher than the insulin. In the same experiment, rosiglitazone was taken as positive control to determine insulin-sensitizing effect on 3T3-L1 adipocytes. The results suggested insulin sensitizing effect on a dose dependent manner for wogonin and norwogonin. Fluorescence absorbance of the wogonin was slightly less than the rosiglitazone at its highest concentration (50 μg/mL), however, norwogonin showed more 2-NBDG uptake than the rosiglitazone at its highest concentration (100 μg/mL). This suggests strong antidiabetic potential for both the monoflavonoid aglycones. Moreover, techtochrysin also exhibited 2-NBDG uptake on matured adipocytes, however, less significant than the wogonin and norwogonin at its highest concentration i.e. 50 μg/mL. Hence, it can be summarised that *T. indica* stems ethanol extract, ethyl acetate fraction, wogonin, norwogonin and techtochrysin exert insulin sensitizing activity in the same manner as rosiglitazone does and therefore are potential antidiabetic substances.

## Conclusion

The present study has shown that wogonin, norwogonin and techtochrysin isolated from *T. indica* stems ethanol extract possess antidiabetic effect on 3T3-L1 adipocytes. Wogonin and norwogonin were found to reveal significant insulin-like and insulin-sensitizing activities at their safe concentrations during adipogenesis. Furthermore, both monoflavonoids displayed significant increase in stimulating 2-NBDG in the absence of insulin. Norwogonin showed higher 2-NBDG uptake activity than the rosiglitazone. This clearly proves insulin-sensitizing ability of these two monoflavonoids. Our study suggests antidiabetic potential of the isolated compounds of *T. indica* in terms of insulin-like and insulin-sensitizing effects. However, we still suggest further in depth research study on the isolated compounds that might lead to the discovery of efficacious antidiabetic drugs.

## Abbreviations

^13^C NMR: ^13^Carbon nuclear magnetic resonance
^1^H NMR: Proton nuclear magnetic resonance
2-NBDG: 2-(N-(7-Nitrobenz-2-oxa-1,3-diazol-4-yl) Amino)-2-Deoxyglucose
ACETONE-D6: Deuterated acetone
APT: Attached proton test
CDCl_3_: Deuterated chloroform
DCM: Dichloromethane
DM: Diabetes mellitus
DMEM: Dulbecco’s modified eagle medium
DMSO: Dimethyl sulfoxide
DPP: Dipeptidyl peptidase
ECM: Extra cellular matrix
EtOH: Ethanol; FBS: Fetal bovine serum
FTIR: Fourier transform infrared spectroscopy
GLUT: Glucose transporter
IBMX: 3-isobutyl-1-methylxanthine
IC_50_: Inhibitory concentration where the response reduced by half
MOA: Mechanism of action
MTT: 3-(4,5-Dimethylthiazol-2-Yl)-2,5-Diphenyltetrazolium bromide
NHMS: National health and morbidity survey
ORO: Oil red O staining
PBS: Phosphate buffered saline
PPARγ: Peroxisome proliferator-activated receptor-γ
PPM: Parts per million
SPSS: Statistical package for the social sciences
T2DM: Type 2 Diabetes Mellitus
TEF: Toluene: Benzene: Formic Acid
TLC: Thin layer chromatography
